# Volumetric study of particle-wake interactions based on free falling finite particles

**DOI:** 10.1007/s00348-025-04066-1

**Published:** 2025-06-29

**Authors:** Yi Hui Tee, James R. Dawson, R. Jason Hearst

**Affiliations:** Department of Energy and Process Engineering, Norwegian University of Science and Technology, Kolbjørn Hejes vei 2, 7050 Trondheim, Norway

## Abstract

Research on free falling particles has predominantly focused on wake dynamics and vortex shedding of individual particles in quiescent flow. However, when these particles fall collectively, the wakes of neighboring particles alter the flow fields. To investigate how the settling and wake dynamics of particles are affected by the wakes of other settling particles, we conducted volumetric experiments using the Shake-The-Box technique. Negatively buoyant 12 mm particles of four different geometries (sphere, flat cuboid, circular, and square cylinders) were first released individually into quiescent water. Subsequently, the particles were released individually into the bulk wakes of 20 monodisperse particles. Using four high-speed cameras and LEDs, we simultaneously captured both 3D particle and fluid motions in the terminal velocity regime. The imaging domain measured 90 mm × 90 mm × 40 mm. Our results show that all trailing particles settling through the bulk wakes gain additional downward momentum from the turbulent wakes, causing them to fall faster than in quiescent flow. However, when the induced velocity of the preceding wakes is subtracted, the relative settling velocity was found to be essentially the same as the particle falling in quiescent fluid. Upstream of the particle, the vortices in the bulk wake interact with the developing shear layer along the particle. The wake downstream of the trailing particle also appears more chaotic than that in quiescent flow.

## Introduction

The dynamics of free falling particles are inherently complex due to their interactions with the surrounding fluid, which can often be chaotic or turbulent. For an individual particle settling in quiescent fluid, most research focuses on how their wake dynamics and vortex shedding are affected by parameters such as density ratio and particle Reynolds number (Ern et al. 2012). While spherical particles have been extensively explored, there is a wide range of non-spherical or anisotropic particles that are poorly understood because of their falling trajectories which are shape dependent (Voth and Soldati 2017). Instead of a steady descent, non-spherical particles may exhibit different settling dynamics such as tumbling, fluttering, or chaotic motion due to instabilities (Willmarth et al. 1964; Field et al. 1997; Ern et al. 2012). These behaviors are significantly influenced by their Reynolds numbers and moments of inertia, which are determined by their shapes and aspect ratios, to name a few (Chrust et al. 2010).

However, when these particles fall in bulk, the flow fields are not only shaped by the wakes of individual particles, but are also perturbed by interactions with neighboring particles. Classical studies such as Fortes et al. (1987) on fluidization have observed how the inertial effects associated with wakes can lead to the capture of one particle in the wake of another. When these particles fall in a confined cell, the particle motions are restricted to 2D motion and any upwash generated from the bulk falling particles will significantly affect the settling particles. Both direct numerical simulation (DNS) by Uhlmann and Doychev (2014) and experimental tracking by Huisman et al. (2016) on the settling of finite spheres with volume fraction of $3\times 10^{-3}$ in a quiescent flow show that when the sphere is undergoing steady oblique descend, they have a higher tendency to cluster strongly in the form of vertically elongated columnar regions (see also Kajishima and Takiguchi 2002). This induces a downward fluid motion that increases the average particle settling velocity. These perturbations, like turbulence, can alter the settling dynamics of the trailing particles.

Previous research on particles in turbulence has focused predominantly on small, heavy inertial particles, where phenomena such as preferential concentration or clustering are prevalent (Maxey 1987; Aliseda et al. 2002; Ferran et al. 2023). For instance, Wang and Maxey (1993)’s DNS using the particle equation of motion with Stokes drag showed that the average particle settling velocity in homogeneous isotropic turbulence is enhanced due to preferential sweeping. Here, the particles tend to migrate toward the downwash regions and hence settle faster. In addition, Huck et al. (2018) showed that at higher particle volume fractions ($10^{-4}$ to $10^{-3}$), instead of preferential sweeping, the velocity enhancement is due to clustering of particles, which generates excess downward forces on regions with high particle density, suggesting the importance of collective effects. For dilute suspension of larger particles, the net increase in the settling velocity decreases due to nonlinear drag effects (see also Good et al. 2014, ). By contrast, for finite-sized spheres with diameters exceeding the Kolmogorov scale where one-way coupling fails and both particle and fluid phases must be fully modeled, Fornari et al. (2016)’s DNS revealed that the mean settling velocity decreases due to strong lateral motions during descent, which amplify nonlinear drag. However, as the particle-to-fluid density ratio increases, the reduction in settling velocity diminishes, with particles following more vertical paths and exhibiting weaker relative lateral velocities.

Among non-spherical particles, disks are one of the most extensively studied geometries, e.g., Chrust et al. (2013) and Tinklenberg et al. (2023). The experimental study by Esteban et al. (2020) on large disks (10–15 mm) falling in water demonstrated that they always fall faster in turbulent conditions. Their Galileo numbers (*Ga*), a measure of the ratio of gravitational to viscous forces acting on a submerged particle, is on the order of $10^4$. The velocity enhancement also increases with the turbulent kinetic energy (or turbulence intensity). Conversely, a recent study by Tinklenberg et al. (2024) found the opposite result, where the disks with *Ga* on the order of $10^1$ to $10^2$ fell slower in turbulent air than in still air. This discrepancy is attributed to the different particle inertia, Galileo numbers, and turbulent conditions that cause the distinctively different falling behaviors of the disks. In the study by Esteban et al. (2020), the disks fluttered while falling. Due to the weaker rotational inertia relative to the fluid, turbulence destabilizes the disks near their turning points, causing them to flutter at higher angles of attack and hence settle faster. Meanwhile, in Tinklenberg et al. (2024), the disks’ rotational inertia is three orders of magnitude larger and they either fell steadily or tumbled. The presence of turbulence induced lateral dispersion, leading to nonlinear drag and slower descent, similar to large spherical particles.

Cylindrical rods and fibers have garnered increasing attention, as most ocean microplastics are found in this shape (Gustavsson et al. 2014; Byron et al. 2015; Giurgiu et al. 2024). For instance, Parsa et al. (2012) and Bordoloi and Variano (2017) focused on the rotational kinematics of cylinders of different aspect ratios in homogeneous isotropic turbulence (see also Oehmke et al. 2021). Meanwhile, recent experiments by Hamidi et al. (2024) demonstrated that a curved cylindrical rod falls faster than a straight cylindrical rod of the same diameter and length in a quiescent flow. This implies that decreasing the curvature of the rod reduces its terminal velocity by increasing the drag coefficient until it matches that of a straight rod. Moreover, the latest parametric study by Bouchet and Dušek (2025) on freely falling cylinders, based on spectral/spectral-element simulation, shows how a flat cylinder transitions from falling with its axis oriented vertically or oscillating, to an oblong cylinder which maintains a relatively horizontal orientation. In summary, these studies underscore that the settling dynamics of both spherical and non-spherical particles are highly dependent on their size, shape, density ratio, and the turbulence scale.

It is important to note that most of the aforementioned experimental studies focused on statistical analysis of the particle dynamics and did not include instantaneous fluid measurements. Nevertheless, due to the complex three-dimensional wake structures around a settling particle, volumetric studies on the flow around falling particles have been limited by experimental challenges. Some advanced measurements that have been implemented include Adhikari and Longmire (2012) who studied the flow around moving objects using tomographic particle image velocimetry (PIV) by Elsinga et al. (2006). In this study, the moving objects were reconstructed separately from the tracer particles using the visual hull technique (Laurentini 1994), which is a silhouette based back-projection for volumetric reconstruction. Meanwhile, Esteban et al. (2019) investigated the flow around free falling polygonal particles using a combination of non-time-resolved volumetric V3V camera systems (Pothos et al. 2009) and separate high-speed camera imaging from two orthogonal directions. Using the recent Shake-The-Box (STB) technique (Schanz et al. 2016), Lorite-Díez et al. (2022) conducted detailed volumetric measurements to investigate the falling paths of different flexible circular cylinders in quiescent fluid due to fluid–structure interaction. The cylinders were reconstructed separately using the tomographic reconstruction (Scarano 2012). More recently, Wieneke and Rockstroh (2024) have studied the flow around a stationary cylinder using the object-aware Lagrangian particle tracking approach. This method incorporates the known shape and position of the object in the form of depth maps to correctly reconstruct tracer particles in the partly occluded regions.

Although extensive study has been conducted to estimate the settling velocity of various particles in both quiescent and turbulent flows, including measurements of actual microplastics particles of irregular shapes (Goral et al. 2023), the effect of wake turbulence on the trailing particles is not well understood. Most of the current studies focus on dilute or single particles in turbulent flow. However, as vortices are shed from finite particle when falling, the wake can alter the flow field and vice versa. For large particles falling en masse, it is difficult to decouple the wake effects from the neighboring particles. Therefore, in this study, we conduct volumetric experiments to investigate how the particle settling velocity and wake dynamics are affected by the wake structures from particles falling collectively. The research goal is to identify particle-wake interactions when both particle Reynolds numbers and Galileo numbers are large, on the order of $10^3$. This is important for the understanding of the transport of large sediments, macroplastic pollutants, or other natural particles in our environment where the particles have been traditionally modeled as point masses in simulations of particle-laden flow at high volume fraction (Brandt and Coletti 2022). The paper is organized as follows. Section 2 discusses the experimental setup, particle parameters, and processing details; Sect. 3 presents result on particle setting velocity in quiescent flow and bulk wake flow, and the three-dimensional vortices shed from the particles. The conclusions are summarized in Sect. 4.

## Methodology

**Fig. 1:**
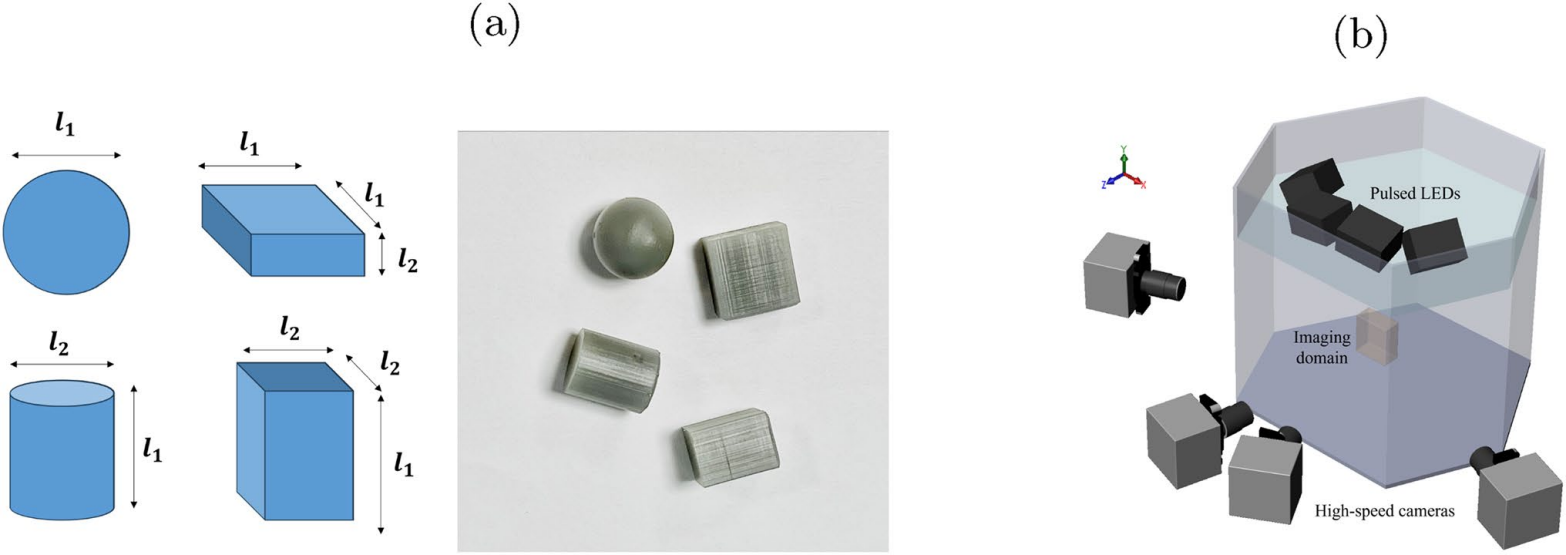
Experimental details. **(a)** Schematic and photograph of $l_1\approx 12$ mm particles. Clockwise from top left: sphere, flat cuboid, square cylinder, and circular cylinder. **(b)** Schematic illustration of the volumetric measurements using four high-speed cameras and LEDs

To investigate the effects of particle shapes on the settling dynamics, we fabricated particles with four distinct geometries but identical mass and volume: a sphere, a flat cuboid, a circular cylinder, and a square cylinder. A Formlabs Form 3 resin printer that has a resolution of 0.05 mm (50 microns) was used to print all the particles. They were printed using Formlabs Tough 1500 resin with a solid-to-fluid density ratio ($\rho _s/\rho _f$) of 1.15. The sphere has a diameter of $l_1\approx 12$ mm, while the other particles have a length of $l_1\approx 12$ mm along their longest axis and a thickness of $l_2$ along their shortest axis. By keeping $l_1$ constant, the values of $l_2$ were chosen such that the volume and mass of each particle matched those of the sphere. Hence, the particle Reynolds numbers, defined as $Re_p=l_1\vert V_p \vert /\nu$, depend solely on the particle velocity ($\vert V_p \vert$). Here, $\nu$ refers to the kinematic viscosity of water. Meanwhile, the particle Galileo number, defined as $Ga=u_gl_1/\nu$ where $u_g=(|\rho _s/\rho _f-1|l_1g)^{1/2}$, is $\sim 1600$ with *g* as the gravitational acceleration. Due to the resin buildup on the side with the smallest area where the supports were printed, both $l_1$ and the particle’s actual mass increase by up to 5.2% and 9% from the targeted values, respectively. Nevertheless, these slight imperfections will not affect the results presented in this current study since all particles were fabricated the same way with high consistency, with standard deviation less than 0.5% and 0.6% in length and mass, respectively, among the printed particles. In addition, no major conclusions were drawn on the rotation dynamics of the particles or the detailed analysis of the wake structures shed from the particles. For statistical and reference purpose, the particle settling velocities in quiescent water were measured using two GoPro HERO 12 cameras arranged in stereoscopic configuration using the particle tracking approach of Muller et al. (2020). Then, the mean settling velocities were computed using the vertical falling velocities of 15 particle trajectories. The detailed parameters of the particles as shown in Fig. 1a can be found in Table 1.

The experiments were conducted in an open hexagonal acrylic tank with dimensions of 0.61 m in length and 0.51 m in width. The tank was filled with water to a height of 0.4 m. To simultaneously capture both the particle dynamics and velocity fields, we set up a time-resolved volumetric measurement system using four Photron FASTCAM Mini WX100 4MP high-speed cameras. The cameras were arranged on the front side of the hexagonal tank, spanning 110$^\circ$, as shown in Fig. 1b. Each camera was equipped with a Sigma 105 mm lens (f/22) and scheimpflugs to enhance focus. Four GSVITEC MultiLED QT pulsed LEDs with 15$^\circ$ lenses were used to illuminate the measurement domain from above. The water was seeded with 40 $\mu$m Dynoseeds. The imaging volume was centered 20$l_1$ below the water surface and 13$l_1$ above the bottom wall to capture steady particle motion while avoiding boundary effects. To prevent particle streaks in the wake regions, depending on the particle settling velocity, the acquisition frequency was varied between 800 Hz and 1000 Hz.

**Table 1:** Particle parameters

Particle	$l_1$ [mm]	$l_2$ [mm]	$Re_p=l_1 \vert V_p \vert /\nu$
Sphere	12.3	–	2640
Circular cylinder	12.6	10.0	1980
Square cylinder	12.6	8.8	1680
Flat Cuboid	12.6	6.3	1500

First, the negatively buoyant particles were released individually into the quiescent liquid below the water surface and allowed to fall with gravity in the negative $y-$direction. To prevent any side wall effects, the particles were released at least 7$l_1$ away from the nearest sidewalls. We waited for at least 20 min before releasing a new particle. These results serve as the reference case. Subsequently, for each particle shape, we released 20 particles into the tank, followed by a trailing particle such that the trailing particle was falling into the bulk wake of the monodisperse particles of identical sizes and shapes. These results were then compared with those from the single-particle falling experiments. For all cases above, the experiments were repeated at least 3 times per case. However, due to the three-dimensional nature of the falling dynamics, some of the particles went out of the field of view during the descent. Among all investigated runs, the trailing particle was between 0.76 s and 3.24 s behind the cloud particles.

For classical multi-camera calibration, a two-level LaVision calibration plate (type 20) was traversed in the *z*-direction of the measurement domain through five positions using a traverse mounted on top of the tank. Then, using DaVis 10.2, a third-order polynomial fit was conducted across all planes to generate the mapping function via the stereoscopic calibration routine. Next, a planar self-calibration routine was performed using 300 images. Here, the images were preprocessed using intensity normalization to normalize the intensity distribution across all cameras. Since the falling particle appeared as a very bright spot in the images as compared to the tracer particles, using the image segmentation algorithm in DaVis 10.2, the big particles (objects) and tracers were separated into two different image sets. With the object masked from the tracer particles, the fluid flow fields were processed using STB (Schanz et al. 2016) followed by the fine-scale reconstruction (VIC#) to convert the Lagrangian particle tracks to Eulerian fields (Jeon et al. 2018). The dewarped image has a size of 1622 $\times$ 1622 pixels that results in a reconstructed domain measuring 90 $\times$ 90 $\times$ 40 mm in the *x*-, *y*-, and *z*-directions, respectively, as shown in Fig. 1b. Using a voxel of 32 pixels, the spatial resolution is equivalent to 1.7 mm or 0.14$l_1$.

Meanwhile, two different methods, tomographic reconstruction and iterative particle reconstruction (IPR), were attempted to reconstruct the falling particles. For the former technique, the segmented images of the large particles were processed using DaVis 10.2 Sequential Motion Tracking Enhancement (SMTE) algorithm to reconstruct their volumes (see Adhikari and Longmire 2012; Lynch and Scarano 2015, for more details). The volumes were reconstructed iteratively in terms of voxel intensities, with 1 signifying the reconstructed object. Similarly, the same images were processed using IPR, which was the particle reconstruction method used in STB (Wieneke 2012). Instead of voxel intensities, this process generates 3D point clouds of the particles, which can be fitted with a sphere or a cuboid to obtain the centroid location for velocity computation.

In this context, using the same computer processing power, IPR demonstrates a significantly higher processing speed compared to tomographic reconstruction using SMTE. For instance, when using IPR, it took only 5 min to generate the point clouds of 480 snapshots of a falling flat cuboid on a high-powered personal work station. In contrast, reconstructing the sequence of volume of the flat cuboid with SMTE took nearly 11 h, making it 132 times slower. Regarding data size, the point cloud reconstruction for this example required just 144 MB of hard disk storage, whereas the tomographic reconstruction produced 3D volumetric data amounting to 25 GB. This indicates that analyzing and plotting the point cloud data are significantly faster than working with the reconstructed image slices. Both techniques rely on the same image sets generated from the segmentation process to reconstruct the particles. In other words, the same calibration was used during the reconstruction process. Therefore, any occlusions caused by uneven lighting or shadows will similarly affect both reconstructions, and both techniques will have similar accuracy based on the calibration and image quality. Given the higher efficiency of IPR, the subsequent analysis of settling dynamics is based on the IPR reconstruction. In this context, the particle centroid positions based on the point clouds were first smoothed by a quintic spline. Based on Schneiders and Sciacchitano (2017), the uncertainties were computed based on the root mean square (r.m.s.) of the difference between the raw and smoothed data. The mean uncertainties of sphere positions (*x*, *y*, *z*) and velocities ($U_p$, $V_p$, $W_p$), obtained by averaging the r.m.s. values over all runs, were 0.7%, 0.8%, and 0.9% of $l_1$, and 0.005, 0.006, and 0.007 ms$^{-1}$, respectively.

## Results and discussion

The four particles investigated (Fig. 1a) have different geometries that affect their falling dynamics in quiescent flow. For both circular and square cylinders, their short aspect ratios led to instability and they tumble as they fall. The square cylinders also exhibit larger dispersion as a result of the spinning behaviors observed. Meanwhile, the flat cuboid flutters, rocks, or oscillates left and right, without tumbling or overturning. The Reynolds number and moment of inertia phase diagram for rectangular plates reported by Smith (1971), plotted similarly to that for circular disks measured by Willmarth et al. (1964), show that when $Re_p=1500$ and dimensionless moment of inertia $I^*=8\rho _s l_2 (l_1^2 + l_2 ^2)/ 3\pi \rho _f l_1^3=0.65$ (see Andersen et al. 2005), the flat cuboid is within the stage of rocking motion. This rocking or fluttering motion aligns well with our observations.

**Fig. 2:**
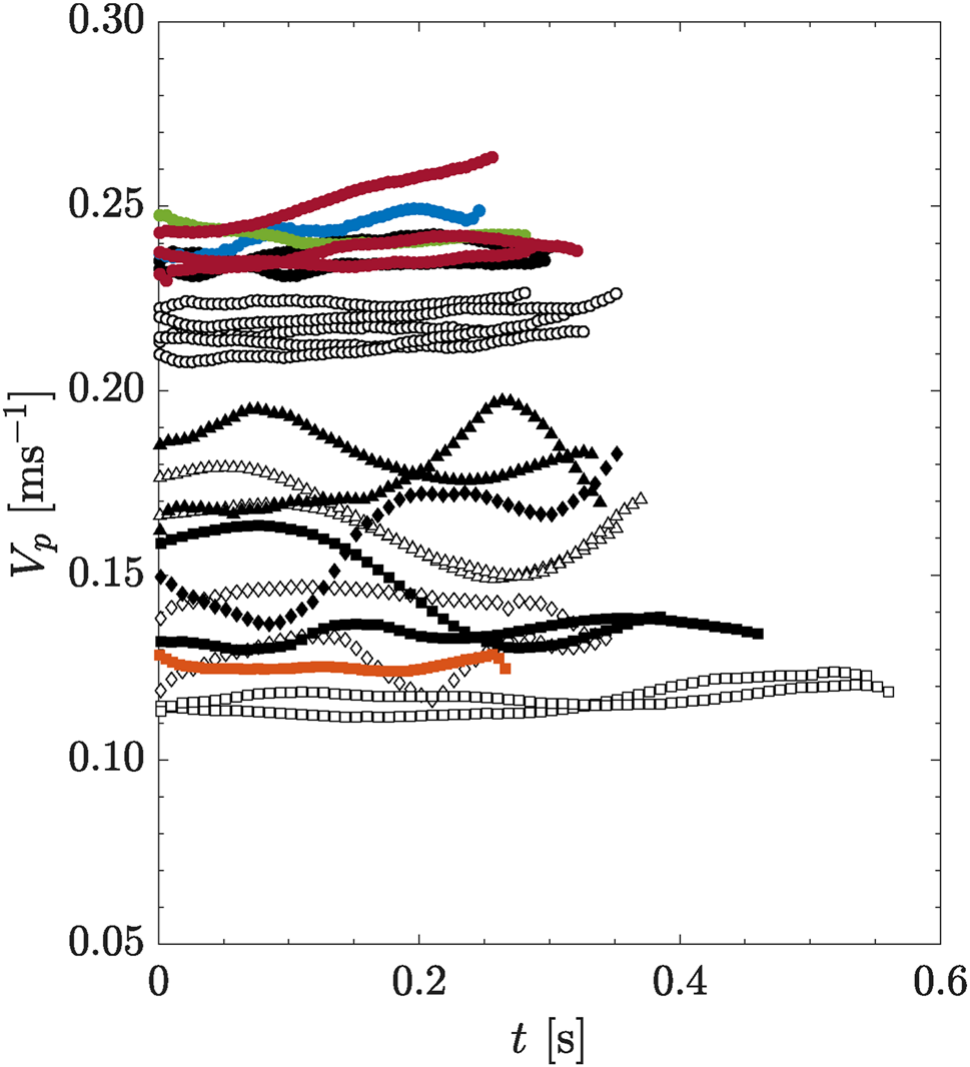
Superposition of particle settling velocity curves from multiple runs, plotted against time. Circle $\bigcirc$: sphere; triangle $\small \triangle$: circular cylinder; diamond $\diamondsuit$: square cylinder; square $\square$: flat cuboid. Empty markers indicate particles falling into a quiescent flow while black filled markers indicate particles falling into the respective bulk wakes of 20 particles. Blue, green, and red circles represent sphere falling into bulk wakes of 20 circular cylinders, square cylinders, and flat cuboids, respectively. Orange markers represent flat cuboid falling in bulk wakes of 20 spheres

Since these particles have equivalent density and mass, their settling velocities in quiescent water are primarily influenced by the drag they encounter while falling, which is due to their different frontal areas. As shown in Fig. 2, the sphere ($\bigcirc$), being more aerodynamically shaped than the other three particles, settles the fastest. The flat cuboid ($\square$) settles the slowest due to the larger drag it experiences. For both cylinders with the same aspect ratio, the rounded surface of the circular cylinder ($\small \triangle$) causes it to sink at a higher velocity than the square cylinder ($\diamondsuit$) with sharp edges. Due to the tumbling motion of the cylinders, their settling velocities also fluctuate more significantly than the sphere and flat cuboids when settling in quiescent water, about 10% of the mean value. This results in $Re_p$ ranging from 1500 to 2640 (see also Table 1).

Based on the particles mean settling velocity ($\overline{V_p}$), we can estimate their mean drag coefficients using the equation of motion, where the drag force equals the net buoyancy force, or $F_D = F_b$, in the vertical direction. Since all particles have the same volume as the sphere, the buoyancy force is given by $F_b = (\rho _s - \rho _f) {\pi l_1^3 g}/{6}$. From this, the calculated mean drag coefficient for the sphere is 0.44, which is close to the value of 0.41 estimated based on Clift et al. (1978). For the flat cuboid, using the surface area of a square, the estimated mean drag coefficient $C_D$ is approximately 1.1, which is at least twice as high as that of a sphere with equivalent mass and volume. Since the flat cuboid falls with the largest frontal area, it experiences the greatest drag compared to the other particles, and thus falls the slowest. The mean drag coefficients for both cylinders are not estimated due to the velocity fluctuations caused by the tumbling behaviors as highlighted above (see also Mougin and Magnaudet 2022).

When comparing the settling velocities in quiescent flow to those of a particle falling into the bulk wake of 20 particles of the same size and shape (as shown by the black filled markers in Fig. 2), we observe that all particles of different geometries sink faster in their respective bulk wake, exhibiting larger vertical velocities. Given that the sphere has the highest settling velocity and is symmetric in shape, we also examined the settling velocities of spheres falling individually into the bulk wakes of 20 circular cylinders (blue markers), square cylinders (green markers), and flat cuboids (red markers), respectively. The results indicate that the spheres fall at approximately the same velocity as when falling into the bulk wakes of 20 spheres (black circles). Since the flat cuboid sinks the slowest, we also analyzed the case where the flat cuboid falls into the bulk wake of 20 spheres (which sink the fastest). The results, plotted with orange markers, show that the flat cuboid also sinks faster than in quiescent flow, and at a rate similar to that of the flat cuboid falling into the bulk wake of the same shape (black square markers). These findings suggest that the geometry of the particles generating the bulk wake has a negligible effect on the settling velocity of the trailing particle.

**Fig. 3:**
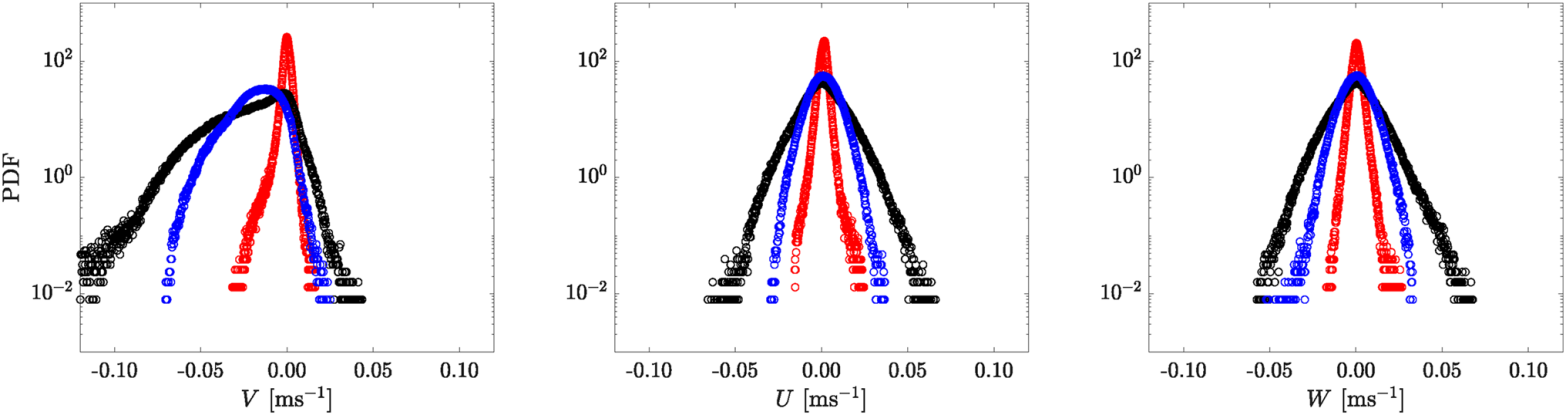
PDF of the three-component fluid velocity for all cases: average of 11 cases right before a particle falling into the quiescent flow enters the top field of view (red), average of 9 cases after 20 collective particles moved entirely out from the bottom field of view (black) and average of 9 cases before a trailing particle falling into the bulk wake enters the top field of view (blue). The time difference between black and blue is within $\sim 2\pm 0.5$ s. Left to right: *V* (vertical velocity), *U* and *W*

To understand the increase in particle settling velocity when falling into bulk wakes, we first evaluate the flow statistics based on the probability density function (PDF) of the velocity within the measurement volume. Figure 3 shows the PDFs for three instances: (1) before the particle enters the top of the measurement volume in a quiescent flow (red); (ii) after all 20 bulk particles exited the bottom of the field of view (black); and (3) before the trailing sphere enters the top of the measurement volume (blue). For (i), we consider all available cases. For (2) and (3), we only consider runs where the time difference between the bulk and trailing particles is within $\sim 2\pm 0.5$ s. Initially, just before the particle enters the top of the measurement volume in a quiescent flow, the PDFs based on 11 runs of quiescent flow (plotted in red) indicate that all fluid velocities (*V*, *U*, and *W* for *y*, *x*, and *z*) are centered around zero with a narrow distribution, suggesting a quiescent-like flow. Conversely, in the bulk particle cases, immediately after all 20 particles exit the bottom of the field of view, the PDFs based on 9 runs (plotted in black) exhibit longer tails, spreading over a wider velocity range. For the vertical velocity (*V*), the collective falling particles induce a stronger downward momentum, causing the PDF to skew toward negative values. Then, just before the trailing particle enters the measurement volume, the negatively skewed *V* (plotted in blue) shifts and centers around $-0.02$ ms$^{-1}$. Comparing this to the velocity increment observed in Fig. 2, we see that they are of a similar magnitude. Meanwhile, the PDFs of *U* and *W* show a similar but narrower distribution than the black, still centered about zero.

**Fig. 4:**
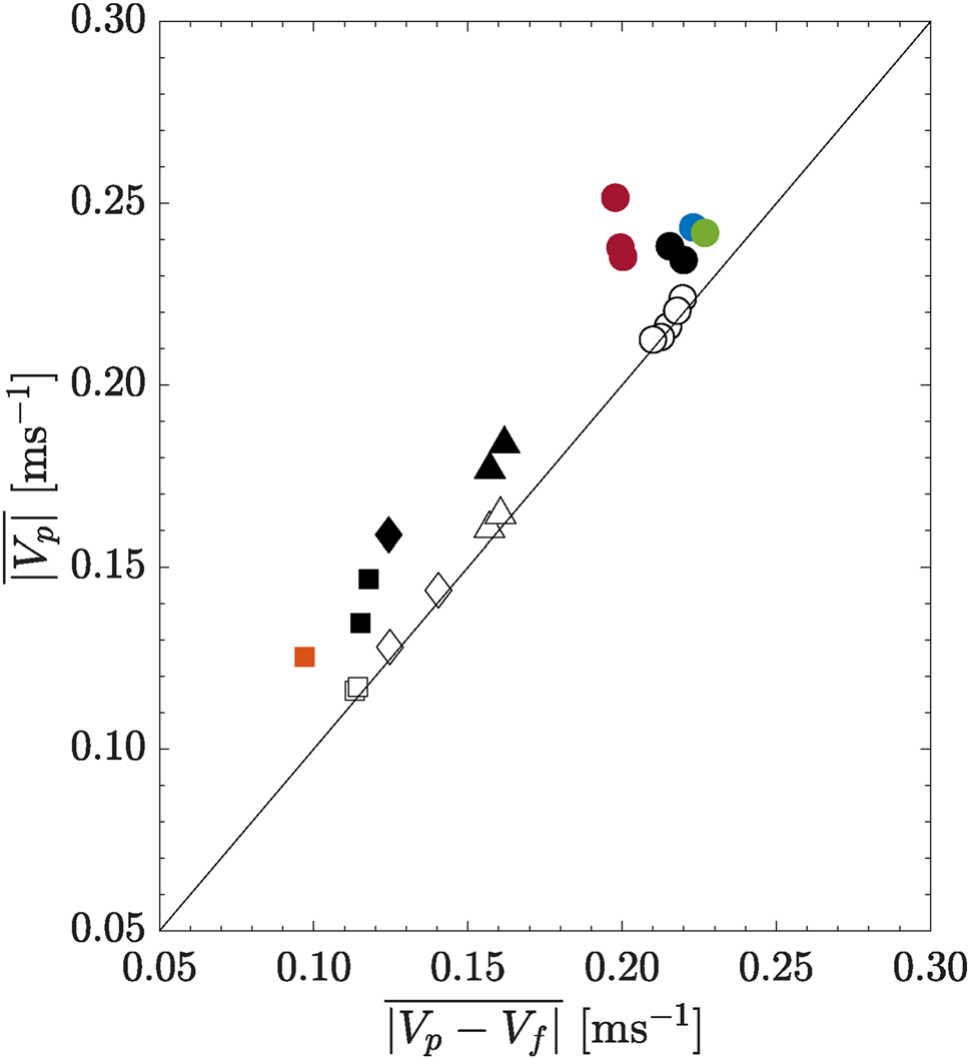
Mean particle settling velocity $\overline{\vert V_p \vert }$ plotted again the mean particle relative vertical velocity $\overline{\vert V_p - V_f \vert }$. Circle $\bigcirc$: sphere; Triangle $\small \triangle$: circular cylinder; Diamond $\diamondsuit$: square cylinder; Square $\square$: flat cuboid. Empty markers indicate particles falling into a quiescent flow while black filled markers indicate particles falling into the respective bulk wakes of 20 particles. Blue, green, and red circles represent sphere falling into bulk wakes of 20 circular cylinders, square cylinders, and flat cuboids, respectively. Orange markers represent flat cuboid falling in bulk wakes of 20 spheres. Straight line represents $\overline{\vert V_p \vert } = \overline{\vert V_p - V_f \vert }$

In this context, since the dominant fluid velocity is in the vertical direction, we compute the particle relative vertical velocity as $V_p - V_f$ where $V_p$ is the particle vertical velocity and $V_f$ is the fluid vertical velocity averaged over the horizontal $x-z$ plane of $d~\times ~d$ (7 $\times$ 7 vectors) at $y=-2d$, or 2*d* upstream of the particle centroid (see also Byron et al. 2019; Tee and Longmire 2024). We then compute the mean relative velocity ($\overline{\vert V_p - V_f \vert }$) for each run. The results of mean particle velocity against the mean relative velocity are plotted in Fig. 4. For all particles falling in the quiescent flow (plotted as empty markers), they fall on the straight line where $\overline{\vert V_p \vert }= \overline{\vert V_p - V_f \vert }$. This indicates that in quiescent flow, since $V_f \approx 0$, the particle is settling due to gravity without any influence from the surrounding fluid. Meanwhile, for all particles falling into their respective bulk wakes (plotted as black markers) as well as bulk wakes of other particles (plotted as colored markers), they fall above the straight line where $\overline{\vert V_p \vert }> \overline{\vert V_p - V_f \vert }$. In other words, $V_f$ is non-negligible. If we consider their relative velocity to that in quiescent flow, the values are similar. (The filled markers are mostly directly above the empty markers.) These results suggest that the higher particle settling velocity in bulk wake is due to the additional momentum from these turbulent wakes and is not necessarily due to some sort of direct drag reduction from the turbulence.

**Fig. 5:**
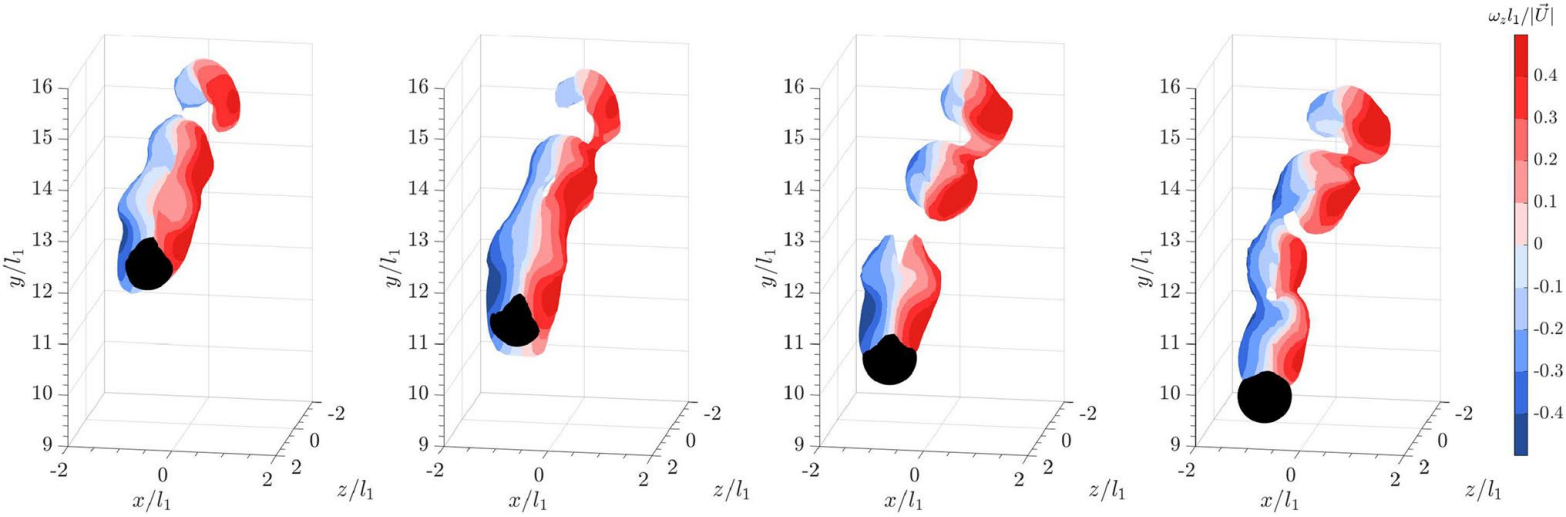
Time series of a sphere falling into a quiescent flow from left to right: isosurface plots of the instantaneous vortical structures visualized using the magnitude of the Lamb vector, $\vert \vec {L} \vert = 0.15$. Color represents spanwise vorticity normalized by sphere diameter and settling velocity in a quiescent flow

**Fig. 6:**
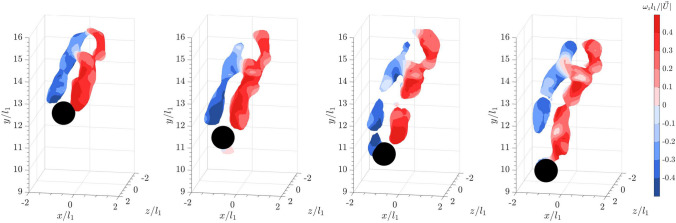
Time series of a sphere falling into a quiescent flow from left to right: isosurface plots of the instantaneous vortical structures visualized using the square of the swirling strength, $\lambda _{ci}^2 = 6$. Color represents spanwise vorticity normalized by sphere diameter and settling velocity in a quiescent flow

Based on the aforementioned examples, we now examine the three-dimensional vortices shed from a sphere in a quiescent flow. Figures 5 and 6 show the isosurfaces of the instantaneous vortical structures visualized using the magnitude of the Lamb vector $\vert \vec {L} \vert$ and the square of the swirling strength $\lambda _{ci}^2$, respectively. The Lamb vector is defined as the cross product of the velocity and the vorticity ($\vec {L}= \vec {u} \times \vec {\omega }$). The Lamb vector appears in the Navier–Stokes equations through the convective acceleration term, which can be decomposed into the kinetic energy term and the Lamb vector term where $(\textbf{u} \cdot \nabla ) \textbf{u} = \frac{1}{2} \nabla (\textbf{u}^2) - \textbf{u} \times \varvec{\omega }$. While the kinetic energy term describes the acceleration of fluid particles due to changes in the kinetic energy across the flow field, the Lamb vector represents the acceleration due to shear and rotational effects (Wu et al. 2006; Hillestad et al. 2024). It illustrates how the velocity field interacts with the vorticity field to produce complex flow features such as vortices and turbulence. As the Lamb vector is a vector field, its magnitude is computed by taking the square root of the sum of the squares of the Lamb vector components. Meanwhile, the swirling strength ($\lambda _{ci}$) plotted in Fig. 6 is derived from the imaginary part of the complex eigenvalues of the local velocity gradient tensor $\nabla \textbf{u}$. It is useful in identifying vortex structures because the imaginary part of the complex eigenvalue quantifies the local swirling motion or rotational strength of the fluid (Zhou et al. 1999). It is a direct indicator of the presence of a vortex, where a higher value of $\lambda _{ci}$ indicates stronger swirling or rotational motion, which is characteristic of vortex cores. The isosurfaces are plotted based on threshold values of $\vert \vec {L} \vert = 0.15$ ms$^{-2}$ and $\lambda _{ci}^2 = 6$ s$^{-2}$, respectively. The color overlay on the isosurfaces represents the spanwise vorticity $\omega _z$, normalized by the sphere diameter and the settling velocity in quiescent flow.

As the sphere descends in quiescent flow, distinct hairpin-like vortices with spanwise vorticity of opposite directions are shed downstream, as illustrated by the isosurfaces of both $\vert \vec {L} \vert$ and $\lambda _{ci}^2$. In this context, dye visualization experiments by Sakamoto and Haniu (1990) demonstrated that for a sphere in uniform flow, when $Re_p>300$, hairpin-shaped vortices begin to be periodically shed from the sphere, forming a laminar wake. As $Re_p$ increases beyond 800, the laminar shear layer starts to shed periodically from the sphere, and the wake flow becomes more turbulent. The direct numerical simulation by Rodriguez et al. (2011) also confirmed that for a sphere within the sub-critical Reynolds numbers of 3700, the instability in the laminar boundary layer separating from the sphere causes the vortex sheets to roll up and transition into a turbulent wake. Consequently, the shed vortices observed in Figs. 5 and 6 resemble the hairpin vortices in a turbulent wake.

**Fig. 7:**
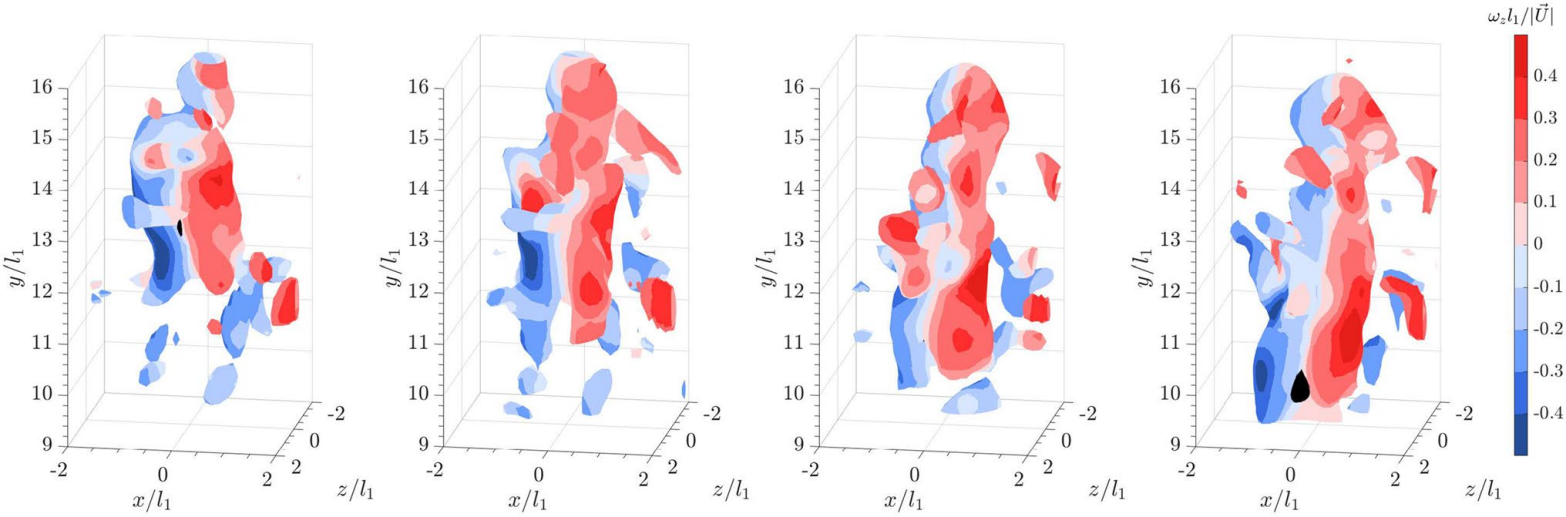
Time series of a sphere falling into the wake of leading collective particles from left to right: isosurface plots of the instantaneous vortical structures visualized using the magnitude of the Lamb vector, $\vert \vec {L} \vert = 0.15$. Color represents spanwise vorticity normalized by sphere diameter and settling velocity in a quiescent flow

**Fig. 8:**
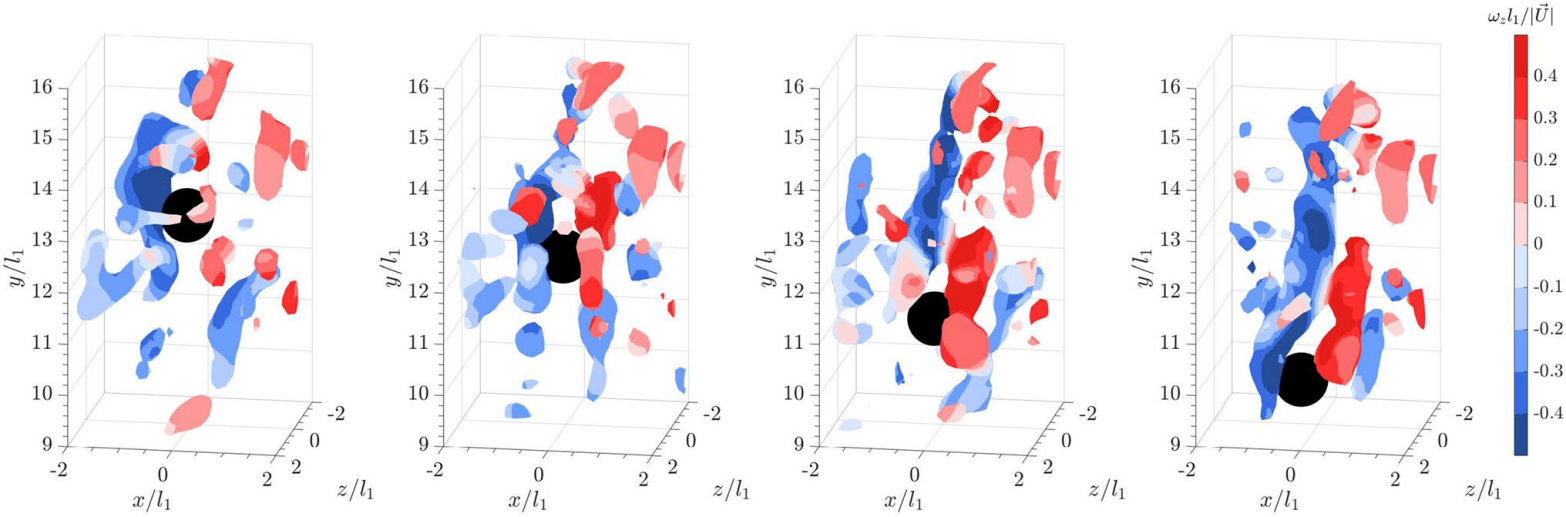
Time series of a sphere falling into the wake of leading collective particles from left to right: isosurface plots of the instantaneous vortical structures visualized using the square of the swirling strength, $\lambda _{ci}^2 = 6$. Color represents spanwise vorticity normalized by sphere diameter and settling velocity in a quiescent flow

For a sphere falling into the bulk wakes of 20 spheres of the same size, the isosurfaces of $\vert \vec {L} \vert$ plotted in Fig. 7 using the same threshold as in Fig. 5 reveal that the sphere is enveloped by cylindrical vortices or vortex tubes starting from almost one diameter upstream of the sphere. The swirling strength plotted in Fig. 8 clearly illustrates that the trailing sphere enters a chaotic region of vortices shed from the leading bulk particles. Compared to the shear layer observed for a sphere in quiescent flow, the upstream vortices are longer and more extended, connecting to the shear layer. When compared to the DNS by Uhlmann and Doychev (2014) on the settling of collective particles, these upstream vortices resemble the columnar structures of the vortices seen in the region of strong downward current, confirming the effects of downwash on the settling of the trailing sphere. Additionally, the distinct hairpin structures observed earlier in Fig. 6 for a sphere settling in quiescent flow are less identifiable here. A similar observation was reported in the DNS by Bagchi and Balachandar (2004) on a stationary, isolated sphere in an isotropic turbulent flow at $Re_p = 610$. They found that the coherent structures in the wake became irregular and dissipated quickly downstream (see also Wu and Faeth 1993). However, it was noted that the downstream shear layers shed from the sphere were instead shortened and broken into smaller vortices. Nevertheless, the results suggest that the turbulent wakes of the leading collective particles interact with the shear layer and disrupt the hairpin vortices shed from the trailing sphere.

**Fig. 9:**
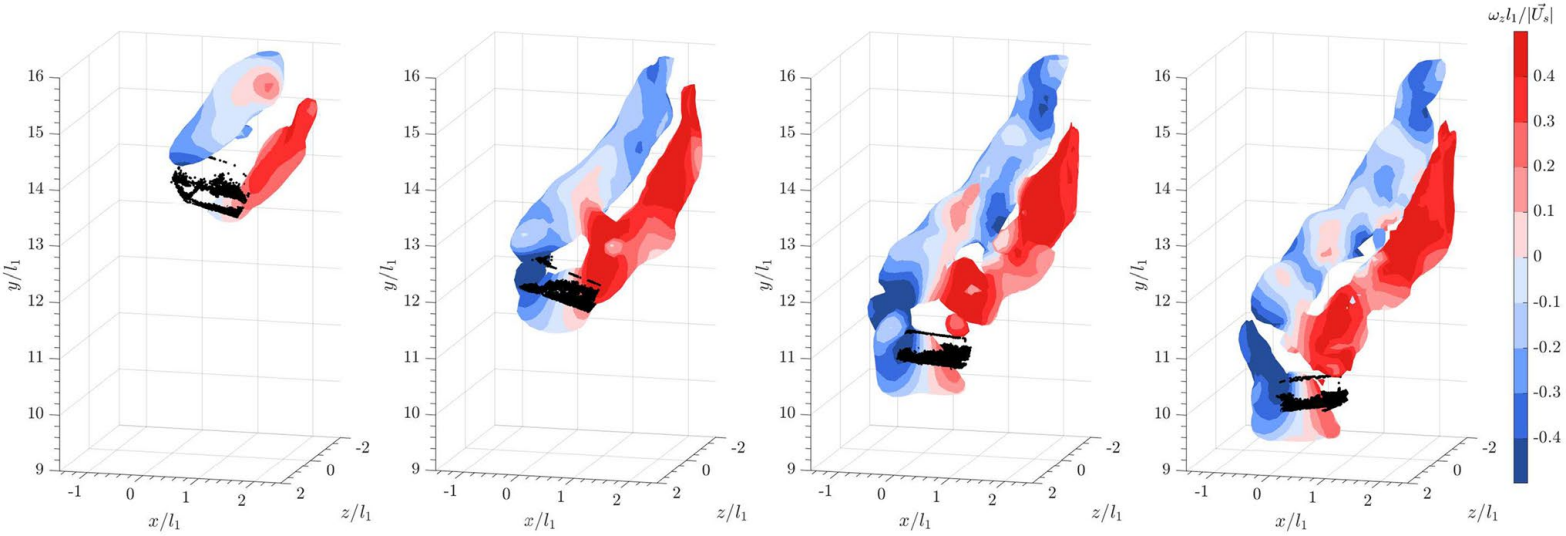
Time series of a flat cuboid falling into a quiescent flow from left to right: isosurface plots of the instantaneous vortical structures visualized using the square of the swirling strength, $\lambda _{ci}^2 = 6$. Color represents spanwise vorticity normalized by sphere diameter and sphere settling velocity ($\vert \vec {U_s} \vert$) in a quiescent flow

For a flat cuboid settling in a quiescent flow, the results in Fig. 9 show that the particle sheds long, streaky structures. The wake also meanders as it falls. As mentioned above, within the reconstructed field of view, the particle oscillates from left to right, without tumbling or overturning.

**Fig. 10:**
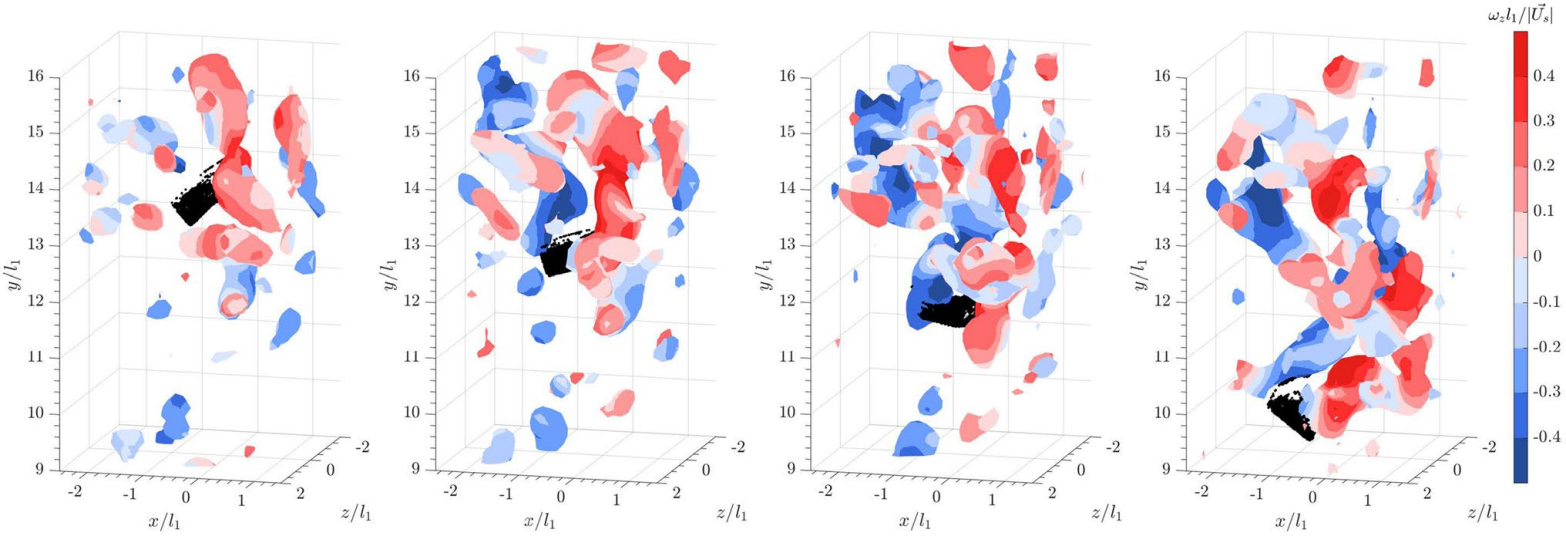
Time series of a flat cuboid falling into the wake of leading collective particles from left to right: isosurface plots of the instantaneous vortical structures visualized using the square of the swirling strength, $\lambda _{ci}^2 = 6$. Color represents spanwise vorticity normalized by sphere diameter and sphere settling velocity ($\vert \vec {U_s} \vert$) in a quiescent flow

When a flat cuboid is released into the bulk wakes, the meandering shed vortices interact with the surrounding vortices (see Fig. 10). Within the measurement volume, this interaction between the bulk wake and the shear layer shedding from the flat cuboid appears to cause the cuboid to flutter more violently from right to left and at a higher amplitude than in quiescent flow.

**Fig. 11:**
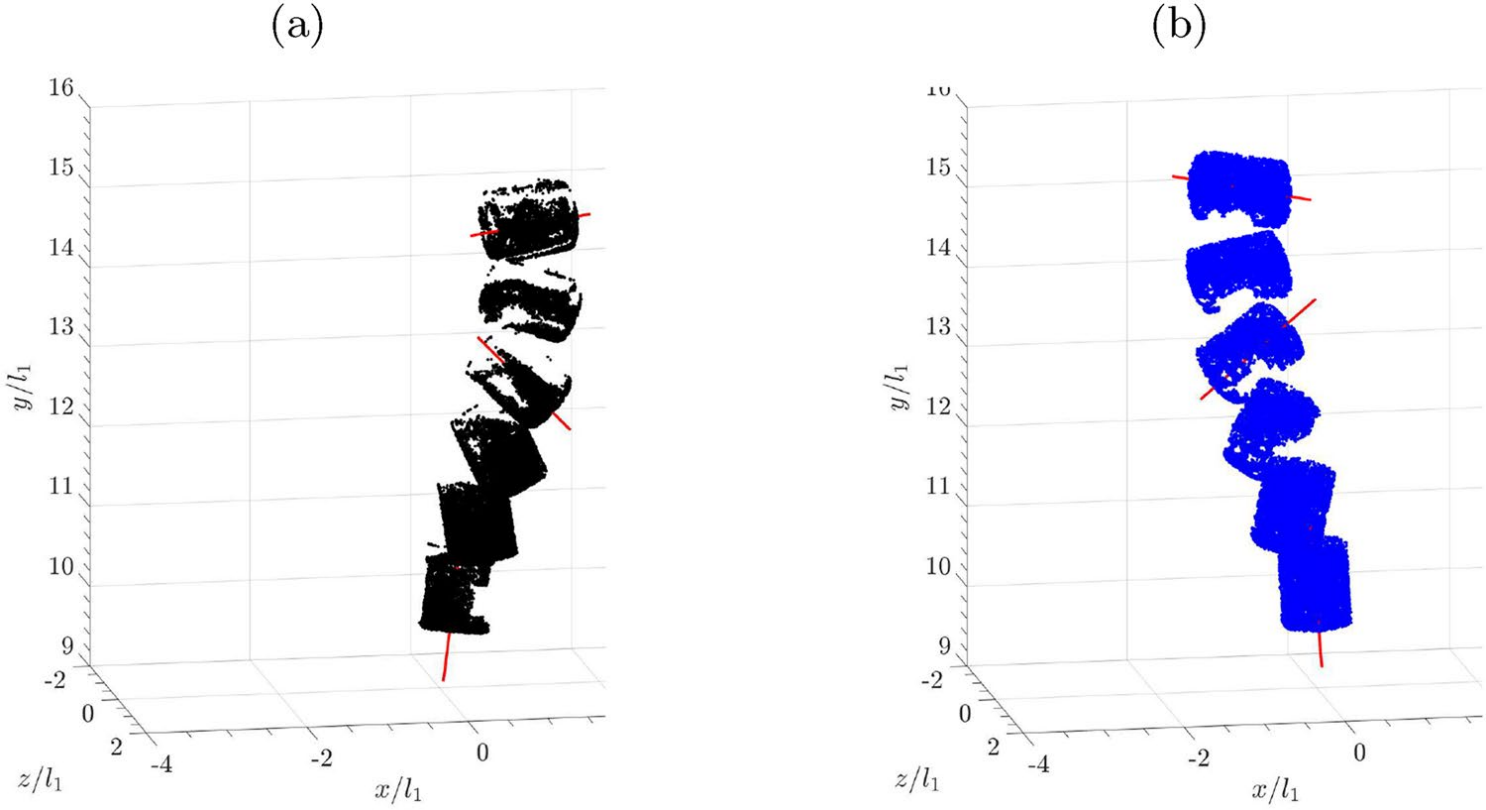
IPR reconstructions of cylinders falling in quiescent flow (**a**) and in the bulk wake (**b**). Red lines indicate the principal axis of the cylinder along its main axis

In general, for the three non-spherical particles, due to their large Reynolds numbers and inertia, their settling dynamics are not significantly altered by the bulk wake. Although the flat cuboid appeared to be fluttering at a larger magnitude, it did not tumble. For the circular cylinder with an aspect ratio defined based on diameter/height or ${l_2}/{l_1} = 0.8$, it tumbled in both quiescent and bulk wakes. Within the field of view as shown in Fig. 11, both settling orientations changed from horizontal to vertical (see also Bouchet and Dušek 2025). This suggests that the tumbling motions are not prompted by the turbulent structures in the bulk wake.

**Fig. 12:**
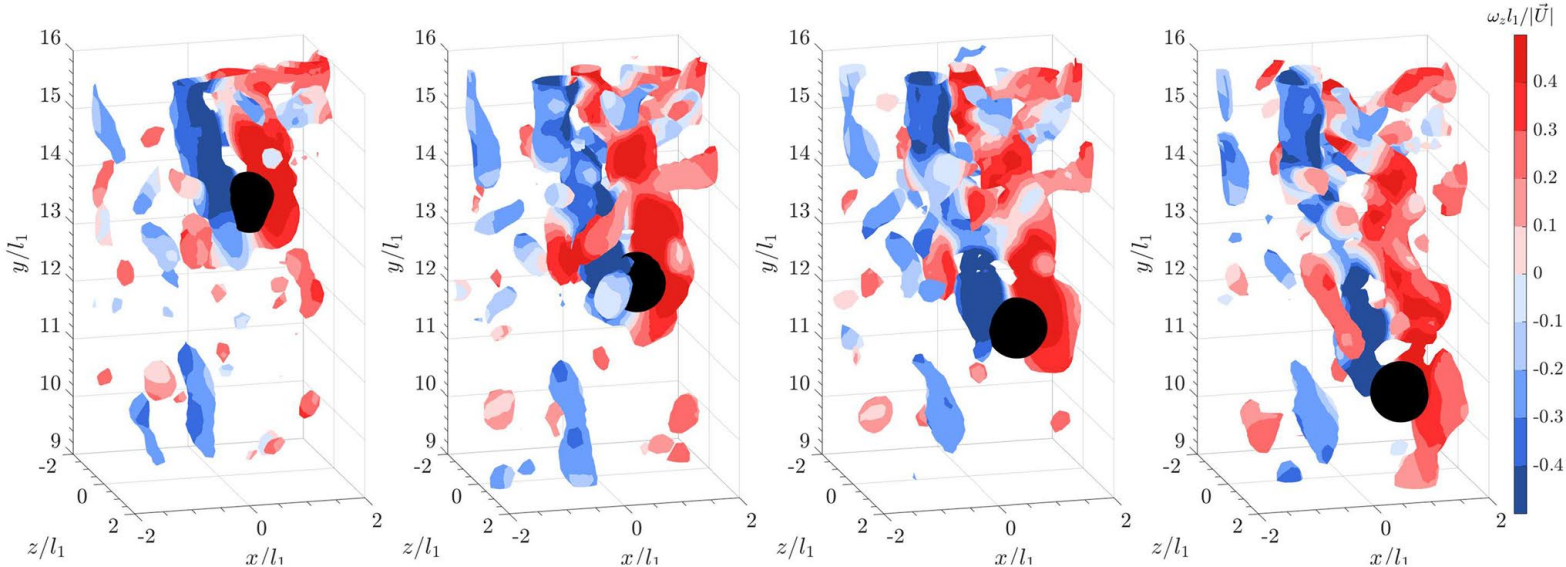
Time series of a sphere falling into the wake of 20 leading flat cuboids from left to right: isosurface plots of the instantaneous vortical structures visualized using the square of the swirling strength, $\lambda _{ci}^2 = 6$. Color represents spanwise vorticity normalized by sphere diameter and settling velocity in a quiescent flow

Finally, when a sphere falls into the bulk wakes of flat cuboids (see Fig. 12), a similar pattern to Fig. 8 is observed, where the shear layer interacts with the upstream vortices. Downstream of the sphere, the hairpin vortex loops are vaguely visible, but the wake is more chaotic. However, as previously mentioned, the interaction of the trailing sphere with various structures of shed vortices does not seem to significantly affect the settling velocity. Since the sphere is shedding within a sub-critical Reynolds number range, where the shear layer transitions from laminar to turbulent (Rodriguez et al. 2011), it is possible that the presence of bulk wakes promotes an early onset of the transition.

## Conclusion

We conducted volumetric experiments to investigate how the particle settling dynamics and shed vortices are affected by the bulk generated by preceding particles falling en masse when both particle Reynolds numbers and Galileo numbers are on the order of 10$^3$. Four high-speed cameras were set up to capture both the settling particle and the surrounding flow fields within the terminal velocity regime. Due to the higher efficiency of iterative particle reconstruction method over the tomographic reconstruction, the former technique was implemented to reconstruct the point clouds of the particles. Simultaneously, the Shake-The-Box technique was implemented to track the flow fields surrounding the falling particles. Four 12 mm particles of different geometries (sphere, circular cylinder, square cylinder, and flat cuboid) were released individually into the bulk wake of 20 collective particles. The results are compared to those in a quiescent flow. All particles of various geometries sink faster in the bulk wakes due to the additional downward vertical momentum induced by the turbulent bulk wakes. Regardless of the shape of the collective particles, the sphere sinks at a similar velocity magnitude in the bulk wakes of the four different particle geometries. Based on the volumetric measurements, the hairpin vortices shed from a sphere at $Re_p=2640$ in a quiescent flow resemble those reported in the literature within the sub-critical Reynolds number range. As the sphere descends into the bulk wake, the upstream vortices left over by the bulk wake due to the downward current interact with the developing shear layer along the particle. Downstream of the sphere, the wake becomes more chaotic. Similar vortex interactions were observed for the flat cuboids, where the fluttering motion caused the wake to meander. Although the particle settling regime for flat cuboids in both quiescent flow and bulk wakes remains unchanged within the phase diagram, the vortex interactions likely cause the flat cuboid to flutter more unstably and with a larger amplitude in the latter.

## Data Availability

All data supporting the findings of this study are available for download at NTNU’s institutional repository hosted by DataverseNO: 10.18710/1RP4OT.
